# Spectral Bidirectional Reflectance Distribution Function Simplification

**DOI:** 10.3390/jimaging11010018

**Published:** 2025-01-11

**Authors:** Shubham Chitnis, Aditya Sole, Sharat Chandran

**Affiliations:** 1 ViGIL, Indian Institute of Technology Bombay, Mumbai 400076, India; shubham.chitnis@iitb.ac.in (S.C.);; 2 Colourlab, Department of Computer Science, Norwegian University of Science and Technology (NTNU), 2815 Gjøvik, Norway

**Keywords:** BRDF, goniochromatic, ANN

## Abstract

Non-diffuse materials (e.g., metallic inks, varnishes, and paints) are widely used in real-world applications. Accurate *spectral* rendering relies on the bidirectional reflectance distribution function (BRDF). Current methods of capturing the BRDFs have proven to be onerous in accomplishing quick turnaround time, from conception and design to production. We propose a multi-layer perceptron for compact spectral material representations, with 31 wavelengths for four real-world packaging materials. Our neural-based scenario reduces measurement requirements while maintaining significant saliency. Unlike tristimulus BRDF acquisition, this spectral approach has not, to our knowledge, been previously explored with neural networks. We demonstrate compelling results for diffuse, glossy, and goniochromatic materials.

## 1. Introduction

**Figure 1:**
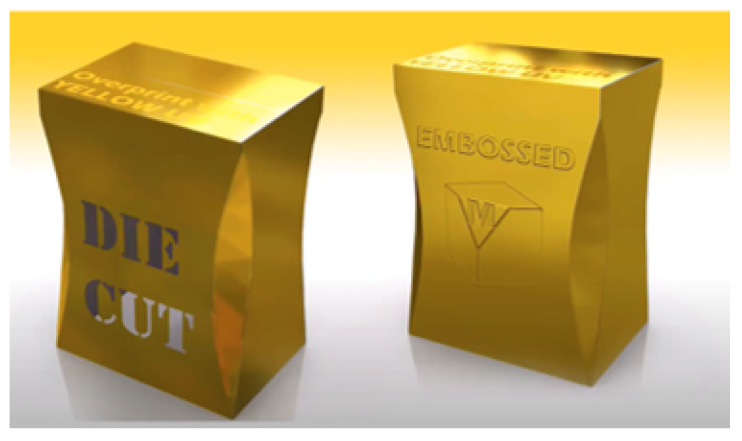
Packaging for fragrance bottles.

Materials with complex optical properties can produce captivating effects in various manufactured products such as paints and specialty coatings, as illustrated in Figure 1.

For instance, they enhance the packaging of cosmetics, alcoholic beverages, and fragrances. These products are produced using different printing techniques and necessitate visual renderings for customer validation and meticulous quality control before production.

**Figure 2:**
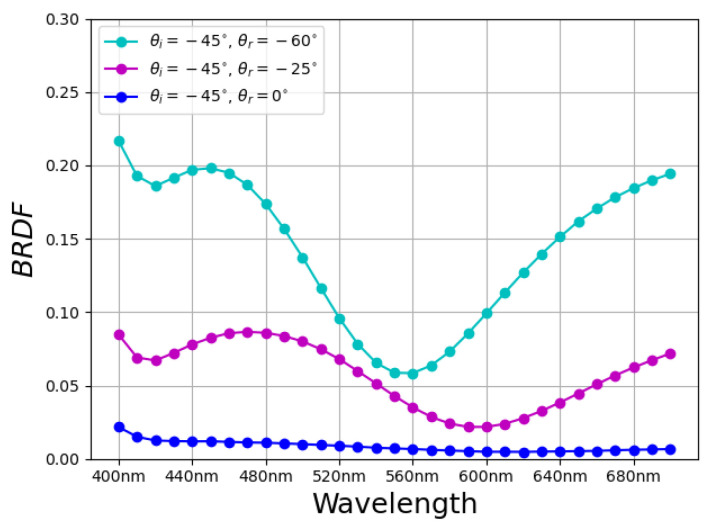
Goniochromatic materials have complex appearances. For example, butterfly wings, oil films, opal, and man-made textiles, such as saris seen in social contexts in India, exhibit this effect. For the same incident angle of illumination ($\theta_{i}=-45^{{}^{\circ}}$), depending on the outgoing angle ($\theta_{r}=-60^{{}^{\circ}}$, $-25^{{}^{\circ}}$ and $0^{{}^{\circ}}$), the measured spectral BRDF of *our* packaging material exhibits vastly different values (note the trough in the $-25^{{}^{\circ}}$ case compared to the $-60^{{}^{\circ}}$). (Angles are in the plane of the surface normal and light directions, and the negative values represent a convention in our measurement setup, which is based on the reference azimuthal angle).

Depending on the illumination (also called incoming in this paper) direction, and reflected (or outgoing) direction, the appearance of these materials can change in terms of perceived lightness or even color (termed goniochromatism, or iridescence). This change is due to the interference in the amount and nature of light reflected (spectrally) from the material in a given direction. Figure 2 shows the spectral bidirectional reflectance distribution function (BRDF) of a goniochromatic packaging print material that we use in this paper. Measuring or modeling the properties of such materials is, therefore, paramount, especially for rapid prototyping.

The surface reflectance of these opaque materials is classically modeled using the BRDF defined by Nicodemus et al. [1] and given in Equation (1).

(1) $$f_{\lambda }\left(l,v\right)=\frac{{dL}_{r}\left(v\right)}{{dE}_{i}\left(l\right)}=\frac{{dL}_{r}\left(v\right)}{L_{i}\left(l\right)\cos\theta_{i}d\omega_{i}}$$

In Equation (1), l and v are incoming and outgoing direction unit vectors, $E_{i}$ is the incoming spectral irradiance, $L_{i}$ is the incoming spectral radiance (flux per unit projected area, per unit solid angle ($\omega_{i}$)) at the incoming angle $\theta_{i}$, $L_{r}$ is the outgoing spectral radiance in the direction *v* out of any of the infinite directions of interest, and *d* is the differential. The unit of a BRDF is the inverse steradian (1/sr), and an explicit reference is made to the wavelength of interest, λ, in the subscript.

The BRDF is used in varied domains such as remote sensing, earth sciences, and, in general, research in optics. From the perspective of rendering, the BRDF was originally modeled in a rudimentary way: the Lambertian model simply used the incoming angle, and the Phong model used a function of the mirror angle and the outgoing angle. These were eminently reasonable for early applications. With increased sophistication, accurate BRDF values for a variety of materials are needed.

The history of the development of BRDF measurement and research is fertile; in the past decade, an average of about 100 publications per year were reported in [2]. Multi-angle, multi-spectral spectrophotometers and gonio-spectrophotometers with mechanical movement have been developed both in the universities and, recently, commercially [3,4,5,6,7]. To reduce the capture time, a camera was employed in 2007 so that multiple outgoing directions are automatically employed, and a theoretical model for “fast BRDF” measurement was shown in [8]. Several other works appeared in quick succession [9,10,11,12,13,14,15,16] culminating in commercialization [16,17,18,19,20,21,22,23,24].

Nonetheless, BRDF measurements—the fodder for rapid appearance modeling—take a considerable amount of time, lack multispectral information (“fast” RGB-camera-based methods), or have difficulty [2] in obtaining a high signal-to-noise ratio outside the specular reflecting area. The measurement times, even with the state-of-the-art approach, can take “2–3 days for anisotropic materials” [25].

**Figure 3:**
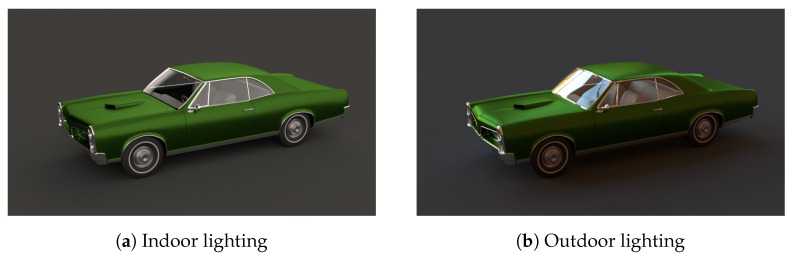
We rendered the *Pontiac GTO 67* scene [28] using the Mitsuba 3 [29] physically accurate rendering engine under different environmental maps [30] to show subtle but important differences in appearance.

A way out is to exploit the inherent redundancy in the BRDF; this has been exploited in RGB rendering-based methods, which use extensive parameterization (for example, reference [26]). However, spectral rendering is needed to preserve details in goniochromatic materials. Additionally, even with “simpler” materials, spectral rendering helps better highlight the differences between indoor and outdoor depictions of the same object. An example showcasing the effect of varying lighting on a material’s appearance is provided in Figure 3. In short, *simulating reality* by solving the fundamental rendering equation [27] is *complete* only with the correct and extensive spectral BRDF data.

In this paper, we keep the focus on rapid prototyping and scientific fidelity and turn to artificial neural networks (ANN) to learn the “missing” BRDF data when performing the measurement is onerous. Indeed, one can either directly regress the final RGB-based appearance from measurements without generating intermediate representations or, more recently, as shown by the authors in [31,32], generate latent representations. These methods take big data and exploit them in novel ways. Being more expressive, neural networks possess the ability to represent a wide range of materials and thus are impervious to model-specific representations.

However, as discussed in Section Neural Nets and BRDF, none of the existing ANN-based methods actually produce spectral measurements. Instead, the goal in the existing works is always to perform the downstream task of processing in the tristimulus domain without explicitly addressing the problem of generating multi-spectral data. The objective of this paper is to, in effect, turn to software measurements, using ANNs to reduce the acquisition cost of *spectral* data.

### 1.1. Contributions

As stated in the title of the paper, we wish to *simplify* the acquisition of BRDF values. Our goal is not to consider downstream tasks such as BRDF editing. Our contributions in this paper can be summarized as follows:

1. We present a multi-layer perceptron (MLP) network for *outputting* the *spectral* BRDF of real-world non-diffuse and (“difficult”) goniochromatic packaging print materials. The MLP by its very nature is more compact and lightweight compared to other neural networks such as transformers or convolutional neural networks, and thus, our scheme saves space and time.
2. We emphasize that, unlike other work, we output multispectral BRDF values while reducing the acquisition cost, and we do not restrict ourselves to rendering.
3. We provide access to the code and spectral measurement data to the scientific community for further research and reproducing the results.

To justify our claims, and for the ground truth, we perform time-consuming measurements (31 wavelengths and a total of 900 incoming–outgoing angle pairs) on four off-the-shelf packaging materials and contrast our software-driven acquisition against the full measurement dataset for a “what-if” comparison.

### 1.2. Prior Work

Since the focus of this paper is on neural simplification, we do not extensively survey the existing types of historical BRDF measurements and refer the interested reader to Section 2 in [2].

#### Neural Nets and BRDF

In one of the early works, [33], a learning-based solution is shown to generate surface appearance. The method uses a single photograph and a self-supervised way of generating more data to model physically plausible spatially varying surface reflectance. This method limits the output to only a spatially varying albedo and homogeneous specular components. Using active lighting with a mobile phone flashlight, better [34,35] specular effects were achieved. In all these cases, the learned neural network has fixed captured input, and thus, the process generalizes the fitting to only these data and their produced output. Multiple captures are required for generating accurate reconstruction. To further improve the state of the art, the authors in [31] take an arbitrary number of inputs and generate a latent space explicitly, which can be taken to be similar to a parametric representation. This can then be subsequently used for generating complex appearances. A similar approach is adopted in DeepBRDF [36], which, however, explicitly uses the MERL [37] database and BRDF measurements. These BRDF measurements are represented as images and fed to a convolutional neural network for obtaining a latent representation; and for BRDF editing, they are back converted to BRDF. This is based on the realistic image synthesis theory of light transport [38], albeit in the RGB domain.

Contrasting with [36] is the work in [39], which also creates a latent representation that can be used further in rendering. The basic neural network (NBRDF) (in [39]), which creates a BRDF, uses a small MLP. The anisotropic materials in [25]—albeit in the RGB domain—can also be modeled. In all of these cases, the emphasis has been on modeling, rendering, and working with tristimulus data.

Motivated by NBRDF [39], we propose an MLP. In contrast to NBRDF, however, we work in the (much larger than 3) spectral domain, with the ultimate goal being the production of measurements rather than BRDF editing or rendering. It is important to note that these MLPs are material-specific, so every material will have a separate trained MLP network.

## 2. Materials and Methods

In this section, we cover some requisite background, followed by our data collection procedure and the proposed MLP network approach.

### 2.1. Reflectance Factor

The BRDF can be theoretically infinite and, therefore, commercially available multi-angle spectrophotometers and goniospectrophotometers [40,41] measure the *biconical reflectance* [38], or simply the reflectance, which is the ratio of the outgoing to incoming flux. Mathematically, with $\theta_{r}$ representing the outgoing angle and $\omega_{r}$ representing the outgoing solid angle, the reflectance is

(2) $$\beta \left(2\pi \to 2\pi \right)=\frac{\int_{\Omega_{r}}L_{r}\left(v\right)\cos\theta_{r}d\omega_{r}}{\int_{\Omega_{i}}L_{i}\left(l\right)\cos\theta_{i}d\omega_{i}}$$

Devices may not output the BRDF or the reflectance per se since they are calibrated with respect to a theoretically perfect reflecting diffuse (PRD) surface. The BRDF is therefore obtained from the ratio relation of the measured values

(3) $$\frac{f_{\mathrm{material}}}{f_{\mathrm{PRD}}}=\frac{\beta_{\mathrm{material}}}{\beta_{\mathrm{PRD}}}$$

with the BRDF of a PRD (denominator on the left hand side) being $\frac{1}{\pi }$. However, in the absence of the theoretical PRD, one can use another surface, such as the Munsell white N9 sheet, which has a reflectivity of 78.66%) [42]. Thus, we calculate the bidirectional reflectance at the material surface by using Equation (4)

(4) $$f\approx \frac{0.79}{\pi }\frac{\beta_{\mathrm{material}}}{\beta_{\mathrm{Munsell}}}$$

where the numerator is the value output by the measurement instrument (GCMS in our case). Once the BRDF is known, the related photometric quantities, such as the radiance $L_{r}$ or the flux, can be calculated.

### 2.2. Data Collection

We describe how we measured our packaging materials. This dataset was used to validate our methods.

#### 2.2.1. Our Packaging Material

Measurement of BRDF for materials with complex reflectance properties can be challenging [25,43,44]. The high dimensionality function with essentially infinite angles can be expensive in terms of time and physical setup required for measurement. A variety of measurement setup designs and methods have been proposed (see [45]).

**Figure 4:**
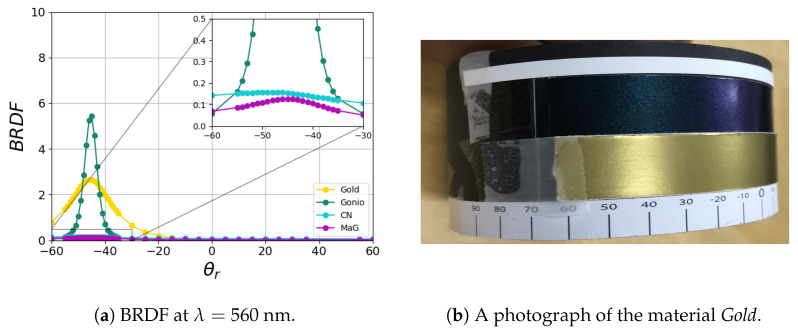
Our diverse packaging materials. Bidirectional reflectance (f) plots for an incoming angle $\theta_{i}=-45^{{}^{\circ}}$ and various outgoing angles $\theta_{r}$.

**Figure 5:**
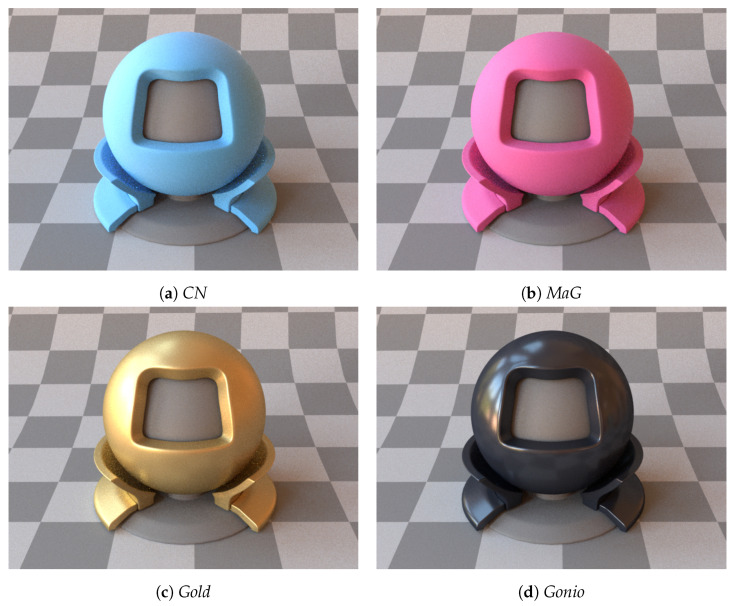
Visualization of the four packaging materials from Section 2.2.

In this work, we provide data for four packaging print materials (named *CN*, *MaG*, *Gold*, and *Gonio*), which are commonly used in the print and packaging industry for decoration and bottle packaging. *CN* (looking like cyan) and *MaG* (looking like magenta) reflect the incident light diffusely, whereas *Gold* and *Gonio* are non-diffuse. *Gonio* is a goniochromatic material that showed a shift in the chromaticity with the change in the outgoing (or viewing) direction, as shown in Figure 2. *Gold* (gold in color) is a thin metallic cardboard commonly used for decoration and bottle packaging, as shown in Figure 4b. Our choice of the four materials is justified in Figure 4a, where the diversity of reflectance properties (diffuse, glossy, and goniochromatic) is exhibited. We also provide a reference visualization using a standard Mitsuba [46] model in Figure 5.

#### 2.2.2. Measurement

**Figure 6:**
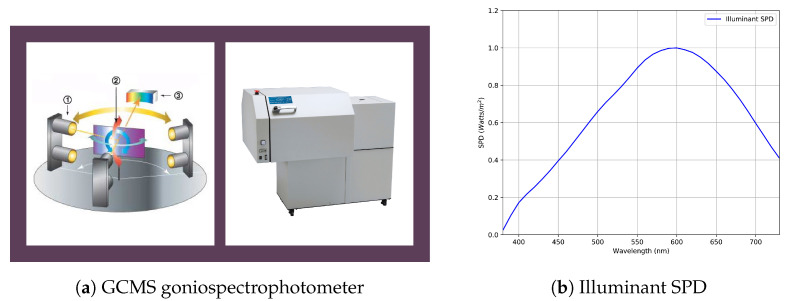
(**a**) The GCMS goniospectrophotometer prototype [https://www.mcrl.co.jp (accessed on 25 November 2024)]. (**b**) Spectral power distribution (SPD) of the point light source.

The BRDFs for the packaging print materials were measured using Murakami’s GCMS-3B goniospectrophotometric color measurement system, henceforth referred to as GCMS. Figure 6a shows a conceptual diagram. An ANSI standard Munsell white N9 sheet was measured along with the packaging print materials and used as reference white to estimate the bidirectional reflectance factor (see Section 2.1) at the material surface. The irradiance at the material surface was estimated using a point light source having a spectral power distribution (SPD) as shown in Figure 6b. GCMS records the spectral radiance factor (390–730 nm at 10 nm intervals) at anormal incoming ($\theta_{i}$) and outgoing ($\theta_{r}$) angles in the range of $+80^{{}^{\circ}}$ to $-80^{{}^{\circ}}$ at $5^{{}^{\circ}}$ intervals. The instrument shows an angular accuracy within $\pm 0.5^{{}^{\circ}}$ and can measure spectrally with wavelength accuracy of ±1 nm at 560 nm and repeatability of ±0.1 nm. Output measurement accuracy is within ±0.5%. It uses a tungsten halogen light bulb to illuminate the materials and a silicon photo-diode array as a detector to detect the outgoing light. The material to be measured rotates between anormal angles $\pm 80^{{}^{\circ}}$ with respect to the incident light source that is normal to the material surface. The instrument provides the radiance factor at the material surface as an output after correcting for the change in illumination and reflected area due to rotation. We calculate the bidirectional reflectance at the material surface using Equation (4), Section 2.1, and the Munsell white N9 sheet with a reflectivity of  79%. For the experiments discussed in this paper, GCMS performs in-plane measurements that take approximately 3 h to measure a single material.

#### 2.2.3. Datasize

Considering the measurement uncertainty at grazing directions for the GCMS [44], measurements recorded outside the range of $+60^{{}^{\circ}}$ to $-60^{{}^{\circ}}$ for both the incoming and outgoing directions were not used in our experiments. The measurement data obtained from the GCMS and used in this study, therefore, consisted of 31 wavelengths (400–700 nm at 10 nm intervals). A total of 900 incoming–outgoing angle pairs are available for 25 unique incoming angles at an interval of 5° with similar outgoing angles, except that the angles around the glossy region are sampled more frequently. When stored in an MLP-training-ready xlsx (Excel) format, the entire dataset, with all the four materials, is sized at approximately 3 MB (770 KB per material).

### 2.3. MLP Network

**Figure 7:**
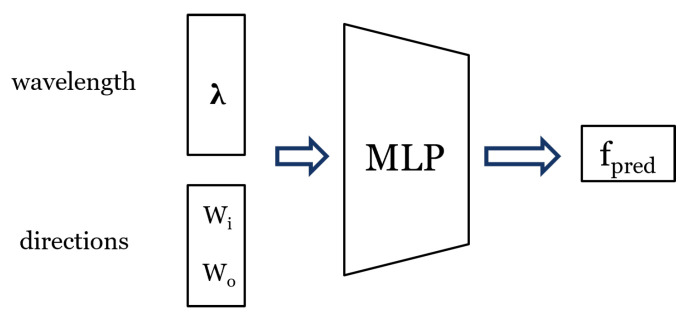
Prototype MLP.

Inspired by NBRDF [39], we suggest the prototype MLP in Figure 7. Variations of this architecture were considered, and the final choice was made based on model performance metrics (loss from Equation (5) for the test dataset in Table 1).

The network takes the incoming and outgoing directions as an input, along with the spectral wavelength (400–700 nm), to predict the sample BRDF as an output.

The network was optimized using the exponential weighted Mean Squared Error (MSE) loss term given in Equation (5). These weights assign higher values in the specular region, pushing the network to capture the specular peaks accurately.

(5) $$L_{\mathrm{MLP}}=\frac{1}{N}\sum_{n=1}^{N}e^{-\gamma \cdot |\theta_{i}^{{}^{\circ}}-\theta_{r}^{{}^{\circ}}|}\cdot {\left(f_{\mathrm{mea}}-f_{\mathrm{pred}}\right)}^{2}$$

In Equation (5), $f_{\mathrm{mea}}$ is the measured sample BRDF, $f_{\mathrm{pred}}$ is the network output, γ is a hyperparameter responsible for modulating the weight assigned to specular predictions, and $\theta_{i}$ and $\theta_{r}$ are the incoming and outgoing directions in degree.

## 3. Results

Despite the complex optical properties of the packaging print materials *Gold* and *Gonio*, in our specific implementation, an MLP network with two 2 hidden layers and 10 nodes each sufficed (NBRDF has 21 hidden nodes and 2 layers in comparison), which takes up a meager 4 KB of space (pytorch binary data). An ADAM optimizer was used with an exponentially decaying learning rate of 0.01. The MLP was trained for a total of 30 epochs, and the weights with the best test accuracy were chosen. For γ, we experimentally determine the value of 3 to be suitable for a generic material, with more specular ones requiring a higher value. The BRDF values measured in Section 2.2 are used as training data for calculating the error signal when training the network. Table 1 shows the distribution of incoming angles for training and testing. It was ensured that the incoming directions covered in the training dataset were not encountered in the test set.

### 3.1. Advantages

We start with our secondary advantage: Unlike a fixed tabular measurement, the neural network can report the BRDF for any unknown angle pair not in the measurements. Our primary result is that instead of measuring *n* incoming–outgoing pairs, we can measure only a fraction *f* of the pairs, thus giving us a measurement time advantage of 100(1−f)%. For example, for the values in Table 1, the total number of angular configurations for the ground truth is 900, and since we hold out 8 incoming angles as the test set with corresponding varied outgoing angles, 293 configurations are not measured. Thus, $f=1-\frac{293}{900}$, and approximately 67% of the acquisition time is saved. On the memory front, our results show that a compact 4 KB MLP network allows us to extrapolate the BRDF values in the test set using approximately 520 KB of BRDF data during training. Comparing this with the full dataset size (per material) of 770 KB (Section 2.2.3), we see that our proposed approach with the neural network does not increase the memory size (in fact, it ends up saving memory in our case). Once the network is trained, we do not need to store the training data. The critical question, therefore, is quantifying the resulting quality lost. To our knowledge, we are the first to employ this line of reasoning for spectral data, and thus, there are no known benchmarks. Nevertheless, we use comparisons from [26] for non-spectral RGB data as described below.

In this section, we show results for the BRDF plots and quantitative errors. Additionally, we provide validation error plots to demonstrate the convergence of the learning process across epochs. We have reserved results for the *CN* and *MaG* samples to the supplementary reading as their diffuse nature makes them easier to learn compared to their specular counterparts.

#### 3.1.1. BRDF Plots

**Figure 8:**
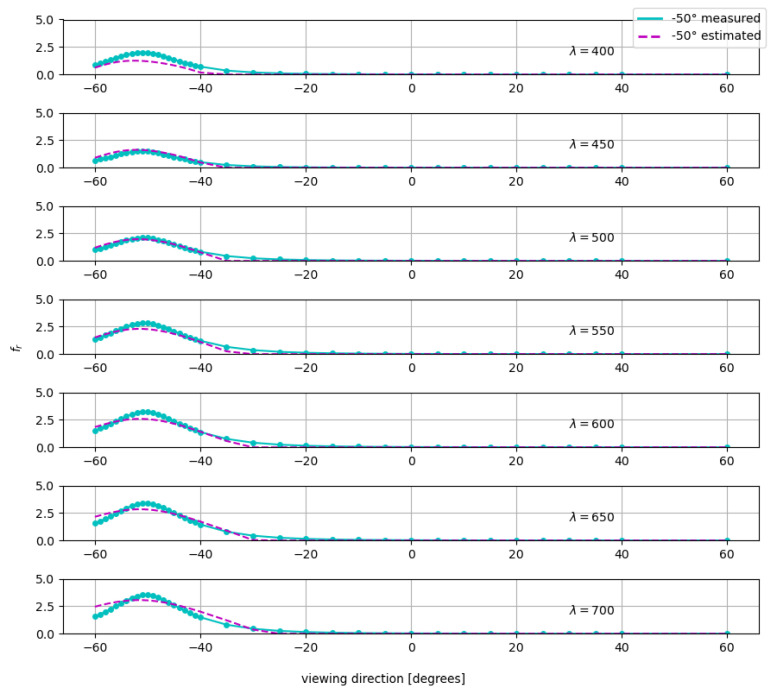
Measured (bold line) and predicted (dotted line) spectral BRDF $f_{r}$, (λ=400,450,500,550,600,650,and700) for the *Gold* packaging sample.

We use the MLP network in the spectral domain to estimate the material BRDF. Figure 8 shows the BRDF for the *Gold* sample for 7 wavelengths and for a single incoming direction of $-50^{{}^{\circ}}$. We show 7 wavelengths across the spectrum only for visual clarity. Results for all wavelengths are in the supplementary material.

From the figure, we see that the network predicts the BRDF measurement data of the sample reasonably accurately and can learn the underlying BRDF trends to extrapolate the unknown BRDF measurement data. This learning is despite the fact that there are only 17 incoming angles to learn from. The network does not produce perfect BRDF in the specular regions, which we attribute to the limited training data. (We considered loss functions that are more forgiving to outliers (L1 loss) and weighing specular samples differently, but the results were similar and provided marginal improvement.)

#### 3.1.2. Quantitative Errors

While the above result is somewhat qualitative, a better sense can be obtained using a relative RMSE error metric (RRMSE), which is calculated using Equation (6).

(6) $$L_{\mathrm{RRMSE}}=\sqrt{\frac{1}{N}\sum_{n=1}^{N}{\left(\frac{f_{\mathrm{pred}}-f_{\mathrm{mea}}}{f_{\mathrm{mea}}}\right)}^{2}}$$

Here, $f_{\mathrm{mea}}$ and $f_{\mathrm{pred}}$ are as defined in Equation (5). For a chosen incoming angle, we calculate this metric for measurements in the glossy region (i.e., where the outgoing angle approximately equals the angle of reflection). In particular, we use a $1^{{}^{\circ}}$ interval for the outgoing angle to closely examine the errors. This is because we expect to see more errors in the glossy region.

**Figure 9:**
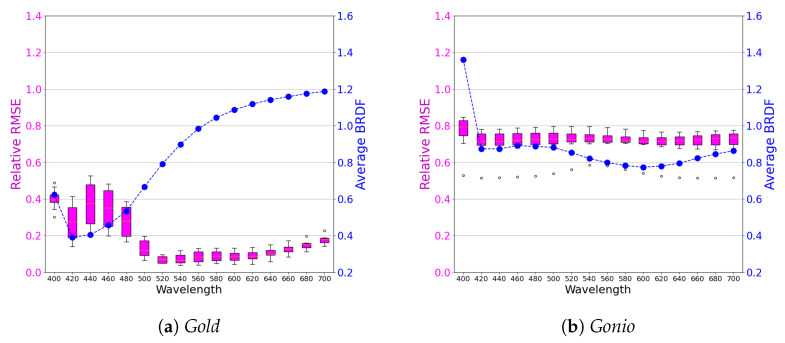
Box-and-whisker plots showing relative RRMSE for *Gold* (**a**) and *Gonio* (**b**) across wavelengths. Only test data (see Table 1) are used to calculate the error metric. The figure also shows (dotted blue) the true measured value of the BRDF; the units in blue are, of course, completely different from the units in magenta. As an example for *Gold*, the network could predict for λ=500 nm the BRDF as 0.7084 instead of 0.7.

To analyze network performance across the spectrum, we provide (see Figure 9) box-and-whisker plots for the relative RMSE metric. Naturally, we only consider errors from the test data, not the training data. Here, for a specific wavelength and for a given incoming direction, all outgoing directions are clubbed together to form a single data point. The box-and-whisker plot thus shows the distribution of errors across multiple incoming directions.

#### 3.1.3. Comparisons

Note that we also show the measured BRDF as a line plot. As an example, consider wavelength 640 nm for the *Gonio* sample. Our predicted value when the measured ground truth expected value is 0.8 corresponds to 0.8±0.71×0.8, i.e., ≈0.85. For the *Gold* sample, picking a random angle of 500 nm, we see that the value of 0.7 could be predicted as 0.7±0.12×0.7, i.e., ≈0.7084.

**Figure 10:**
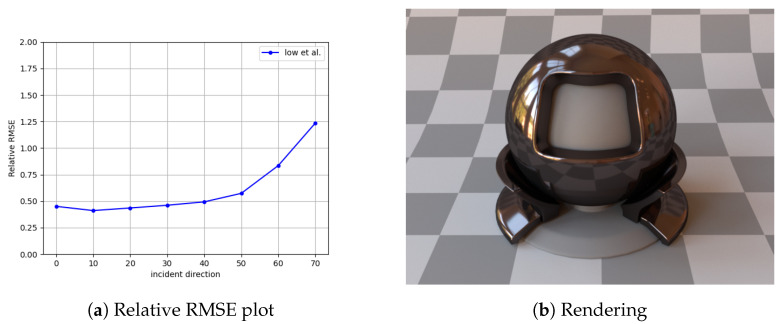
(**a**) Relative RMSE error plots for *Tungsten Carbide*, which are similar to Figure 9. (**b**) *Tungsten Carbide* rendered using Mitsuba.

As mentioned earlier, spectral simplification along the lines we have described has not been performed. However, for RGB data, we have the results from, for example, [26]. Here, a parametric model is employed on RGB data from MERL [37] with about 28,800 outgoing rays per incident angle. Thus, approximately 230,400 samples (i.e., 256 times our value of 900) have been employed in pruning BRDF data. The question at hand is to compare and contrast the corresponding loss in quality. In the case of a randomly chosen MERL material, Figure 10 shows a relative RMSE plot for the chosen MERL material along with a typical rendering with data from [26]. The mean RRMSE turns out to be 0.61, and thus, our RRMSE Figure 9 is quite comparable to the state of the art in our spectral implementation.

### 3.2. Convergence Plots

**Figure 11:**
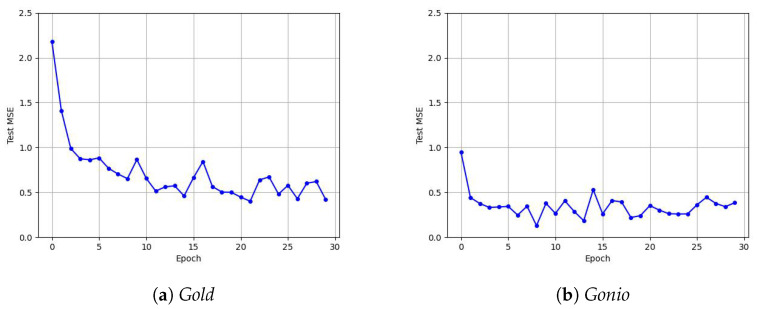
Mean squared error plots on the test set as the MLP training progresses.

For the chosen MLP architecture, we plot test errors (Figure 11) written across different epochs. This is the mean squared error (MSE). As per standard practice, plotting losses from Equation (5) (i.e., the term being optimized) is not recommended since it is biased in our favor. One can observe that the loss diminishes steadily within the first 10 epochs, following which we see small variations as the training progresses.

## 4. Discussion

BRDF simplification (for RGB rendering) post measurement can be performed with parametric models. Parametric models are built with very strong domain priors, which allow them to learn the underlying physics with significantly less data storage compared to the neural networks. While parametric models are more efficient, they are limited by the accuracy of the underlying theory. There may be limited applicability to a parametric model, and something like the ABC model [26] cannot be directly applied to a different range of materials (like anisotropic materials). Classical mathematical tools such as Spherical Harmonics or Tensorial Decomposition [47] can be employed to interpolate or extrapolate data, but they require fixed handcrafted parameters compared to learned parameters in ANN. Nevertheless, though neural networks present an interesting approach to data approximation, their data-hungry nature limits their abilities. The strength that networks bring is more in terms of the offered flexibility. Our experiments indicate their feasibility.

## 5. Conclusions

Packaging objects, such as the example described in Figure 1, or obtaining the “right” shade for car paints, calls for varying the material, measuring the BRDF, and repeated rendering. Rapid prototyping is a modern trend with international supply chains and calls for a quick turnaround of the entire process with new paints and coatings. Given that spectral data are richer than their tristimulus counterpart, and given that existing analytical models are being restricted to the RGB space, multi-layer perceptron networks were proposed for spectral BRDF estimation in this paper. These networks learn the underlying patterns in the BRDF data and provide suitable approximations, which can be used either for rapid prototyping or for scientific experiments. This approach can offer a significant reduction in the required measurement data and, thus, acquisition time (approximately 67% in our experiments). To the best of our knowledge, no previous work addresses this gap in the scientific know-how in the spectral domain.

All our conclusions are based on visual and quantitative ablation studies.

## Figures and Tables

**Table 1:** Consideration of angles used to train the ANN.

Dataset	Incoming Angles ($\theta_{i}^{{}^{\circ}}$)
Train	{$-60^{{}^{\circ}}$, $-55^{{}^{\circ}}$, $-45^{{}^{\circ}}$, $-40^{{}^{\circ}}$, $-30^{{}^{\circ}}$, $-25^{{}^{\circ}}$, $-15^{{}^{\circ}}$, $-10^{{}^{\circ}}$,
	$0^{{}^{\circ}}$, $5^{{}^{\circ}}$, $15^{{}^{\circ}}$, $20^{{}^{\circ}}$, $30^{{}^{\circ}}$, $35^{{}^{\circ}}$, $45^{{}^{\circ}}$, $50^{{}^{\circ}}$, $60^{{}^{\circ}}$}
Test	{$-50^{{}^{\circ}}$, $-35^{{}^{\circ}}$, $-20^{{}^{\circ}}$, $-5^{{}^{\circ}}$, $10^{{}^{\circ}}$, $25^{{}^{\circ}}$, $40^{{}^{\circ}}$, $55^{{}^{\circ}}$}

## Data Availability

The measured BRDF data, the trained MLP network, and the optimized hyperparameters can be accessed using the following link. [https://shubham3008.github.io/spectral-simplification-release] (accessed on 11 September 2024).

## Supplementary Materials

The following supporting information can be downloaded at: https://www.mdpi.com/article/10.3390/jimaging11010018/s1, Figure S1: Measured and Predicted BRDF plots for 31 wavelengths (*Gold* sample); Figure S2: Relative RMSE box-and-whisker plots (*CN* and *MaG* samples); Figure S3: MSE convergence plots (*CN* and *MaG* samples).

## Abbreviations

The following abbreviations are used in this manuscript:

BRDF	Bidirectional Reflectance Distribution Function
ANN	artificial neural network
MLP	multi-layer perceptron
MSE	Mean square error
RMSE	Root MSE
RRMSE	relative RMSE
