# Supplementary material for: Spectral Bidirectional Reflectance Distribution Function Simplification
Source: J Imaging. 2025 Jan 11;11(1):18. doi: 10.3390/jimaging11010018 (PMC11765975; doi:10.3390/jimaging11010018)
Supplement: Supplementary file 1 [file jimaging-11-00018-s001.zip › jimaging-3366035-supplementary.pdf]

Spectral BRDF Simplification: Supplemental Document

1. VISUAL PLOTS

Similar to the paper (Section 3.1.1), we have BRDF plots across wavelengths for the packaging print material *Gold* (Figures S1).

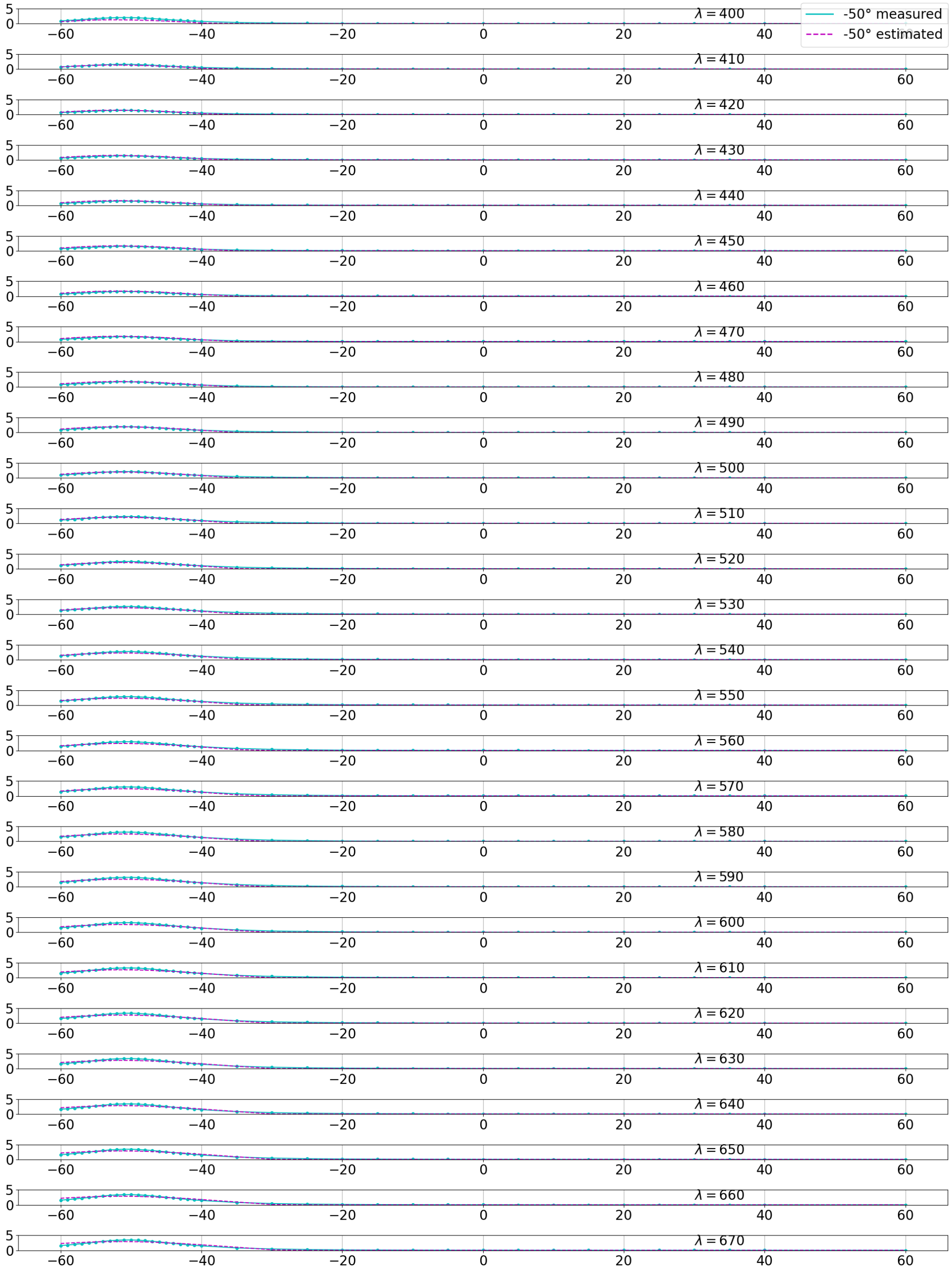

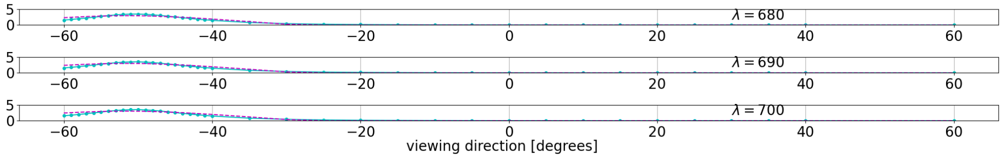

Figure S1. Measured and predicted spectral BRDF for 31 wavelengths of the *Gold* packaging material. The incident angle chosen was $\theta_i = -50°$.

2. QUANTITATIVE PLOTS

We follow the relative-rmse error metric and get a spectral boxplot for the packaging print materials (Figure S2).

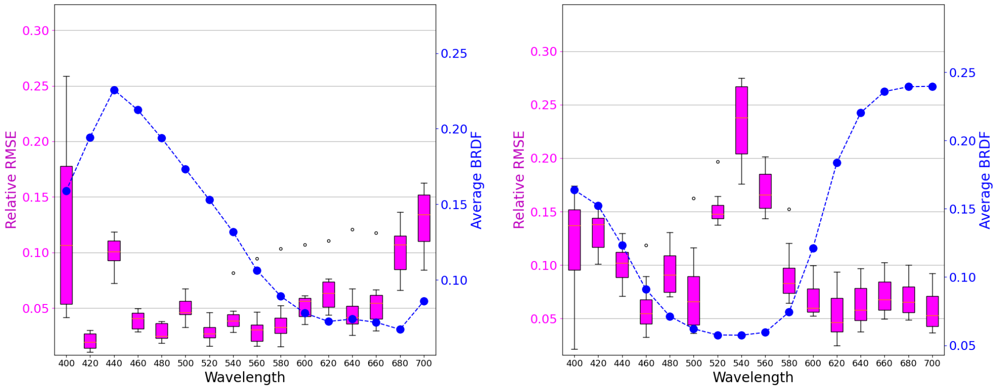

(a) *CN* (b) *MaG*

Figure S2. Box-and-whisker plots showing relative RMSE for the packaging print materials *CN* (left) and *MaG* (right) across wavelengths. Only test data (see Table 1 in the main paper) is used to calculate the error metric.

3. CONVERGENCE PLOTS

During MLP training, mean squared error plots are generated for the *CN* and *MaG* materials on the test set (Figure S3) similar to Section 3.2 in the main paper.

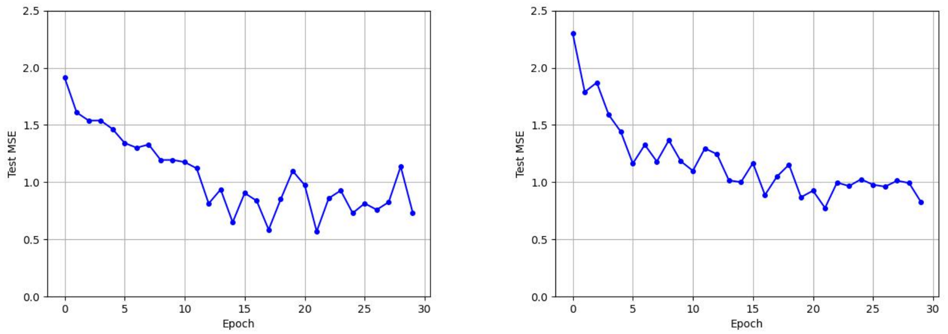

(a) *CN* (b) *MaG*

Figure S3. Mean squared error plots on the test set as the MLP training progresses for the packag-ing print materials *CN* (left) and *MaG* (right). Only test data (see Table 1 in the main paper) is used to calculate the error metric.
